# The Role of Myositis-Specific and Myositis-Associated Autoantibodies and the Activation of Type I Interferon Pathway in the Generation of Clinical Phenotypes of Inflammatory Myopathies

**DOI:** 10.31138/mjr.34.2.275

**Published:** 2023-06-30

**Authors:** Charalampos Skarlis, Nikolaos Michalakeas, Maria Gerochristou, Sylvia Raftopoulou, Nikolaos Marketos, Kyriaki Boki, Dimitrios Vassilopoulos, Alexandros Panayiotis Stratigos, Dimitrios Boumpas, Clio Mavragani

**Affiliations:** 1 Department of Physiology, Faculty of Medicine, National and Kapodistrian University of Athens, Athens, Greece,; 2 First Department of Dermatology and Venereology, Faculty of Medicine, National and Kapodistrian University of Athens, Athens, Greece,; 3 Sismanogleio General Hospital, Athens, Greece,; 4 Clinical Immunology-Rheumatology, Hippokration General Hospital, Faculty of Medicine, National and Kapodistrian University of Athens, Athens, Greece,; 5 Joint Academic Rheumatology Program, Athens, Greece,; 6 Faculty of Medicine, National and Kapodistrian University of Athens, Athens, Greece,; 7 Fourth Department of Internal Medicine, University Hospital Attikon, National and Kapodistrian University of Athens, Athens, Greece

**Keywords:** inflammatory myositis, type I interferons, autoantibodies

## Abstract

Idiopathic inflammatory myopathies (IIMs) are a group of heterogeneous autoimmune diseases with a prevalence of 20 cases per 100000 of population. Despite their diversity, IMMs are characterised by several common clinical features such as muscle inflammation, proximal muscle weakness, abnormal electromyography and/or muscle biopsy. Over the last years, it has been increasingly recognised that an array of autoantibodies known as myositis-specific antibodies (MSAs) and myositis-associated antibodies (MAAs) are associated with distinct clinical phenotypes and diverse prognosis. Although the exact underlying mechanism of IIMs is not fully understood, accumulating data suggest that the activation of type I interferon pathway plays a central role in disease development. Previous studies have reported the upregulation of type I interferon (IFN) induced genes in peripheral blood and muscle biopsies derived from myositis patients. Given the heterogeneity of inflammatory myopathies along with the central role of type I IFN pathway in disease pathogenesis, the aim of the current study is to elucidate the link between distinct clinical phenotypes of inflammatory myopathies with the presence of serum MSAs or MAAs, as well as with type I IFN activation.

## INTRODUCTION

Idiopathic inflammatory myopathies (IIMs) are a group of heterogeneous autoimmune diseases with a prevalence of 20 in 100,000. Despite their diversity, they present numerous common clinical features including muscle inflammation, proximal muscle weakness, elevated serum muscle enzyme values and abnormal electromyography and/or muscle biopsy.^1–3^ Other organs may be also affected, such as skin, joints, respiratory, cardiovascular system, and gastrointestinal tract. Over the last years, it has been increasingly recognized that an array of autoantibodies known as myositis-specific antibodies (MSAs) and myositis-associated antibodies (MAAs) are associated with distinct clinical phenotypes and diverse prognosis.^4–7^ Although the exact underlying mechanism of IIM’s is not fully understood, accumulating data suggest that the activation of type I interferon pathway plays a central role in disease development.^8–10^

Interferons (IFNs) are functionally related cytokines of innate immunity, exerting antiviral, antimicrobial, antitumor and immunomodulatory activities (9). IFNs display a well-known antiviral action, however it is well appreciated that they contribute to the chronic inflammatory process in organ-related and systematic autoimmune diseases as well.^11–13^ There are three main classes of interferons, type I, type II and type III interferons. Type I is constituted by IFNα, β, δ, ω, ɛ, τ, ω, ζ, λ, and κ, with the first two being the most well-studied. Almost all nucleated cells can produce interferons, however their main producer are plasmacytoid dendritic cells (pDCs).

Physiologically, type I IFN secretion is mediated by the activation of pattern recognition receptors (PRRs). Pathogen-associated molecular patterns (PAMPs) such as microbial lipopolysaccharide (LPS) and endogenous or exogenous nucleic acids are sensed by PRRs such as the cell surface and endosomal membrane-bound toll-like receptors (TLRs), binding LPS and DNA or RNA respectively.^14^ Additionally, the cytosolic retinoic acid-inducible gene 1 (RIG-I)-like family of receptors^15^ along with the melanoma differentiation-association protein 5 (MDA5) are RNA sensors, while the cyclic GMP-AMP synthase (cGAS) detects DNA, leading to activation of interferon stimulatory genes protein (STING) and type I IFNs production.^9,16^

Once produced and released to circulation, type I inter-ferons bind to IFNα/β receptor (IFNAR) leading to auto-phosphorylation and activation of the Janus kinase (JAK)-signal transducer and activator of transcription (STAT) pathway. The latter binds to IRF9, leading to the formation of IFN-stimulated gene factor 3 (ISGF3). ISGF3 acts as transcription factor positively regulating the transcription of hundreds of interferon-stimulated genes (ISG).^11,17^

Previous studies have reported an upregulation of type I interferon inducible genes in peripheral blood^18^ and muscle biopsies^19^ derived from myositis patients. Particularly, the overexpression of interferon induced gene 15 (ISG15) and the nucleic acids receptors RIG-1 and TLR-3 in myositis patient’s muscle biopsies has been associated with chronic inflammation which ultimately was shown to lead to muscle atrophy.^11,20^ This association is further supported by data derived from *in vitro* studies suggesting that type I IFNs inhibit myoblast differentiation while inducing the atrophy of muscle tubules in vitro. Furthermore, type I IFNs cause mitochondrial malfunction and inflammation in dermatomyositis patients.^8^ Additionally they enhance the production of B-cell activating factor (BAFF) which contributes to autoantibody production through differentiation of B-cells to plasma cells.^20^

## AIM OF THE STUDY

Given the clinical and laboratory heterogeneity of inflammatory myopathies along with the central role of type I IFN pathway in disease pathogenesis, the aim of the current study is to elucidate the link between distinct clinical phenotypes of inflammatory myopathies with the presence of serum MSAs or MAAs, as well as with type I IFN activation.

## PATIENTS AND METHODS

Peripheral whole blood will be obtained from patients referring to the Molecular and Applied Physiology Unit at the Medical School of National and Kapodistrian University of Athens for evaluation of MSAs or MAAs. Participating referral centres include First Department of Dermatology and Venereology, “Andreas Syggros” Hospital, Rheumatology Unit, Sismanogleio General Hospital, Clinical Immunology-Rheumatology Unit, 2nd Department of Medicine and Laboratory, Hippokration General Hospital and Fourth Department of Internal Medicine, School of Medicine, University Hospital Attikon, NKUA,12462 Haidari, Greece.

Demographics, clinical, laboratory and histopathological features will be recorded, after consultation with the referring physician. More specifically, skin manifestations (oral ulcers, puffy hands, V-sign rash, Gottron’s papules and inverse Gottron, mechanic’s hands, periungual erythema, heliotrope sign, facial erythema, flagellate erythema, shawl sign, vesiculobullous eruptions, calcinosis cutis, livedo reticularis, purpura, alopecia, panniculitis, skin ulcers, poikiloderma), musculoskeletal features (arthralgias, morning stiffness, arthritis, myalgias, muscle weakness (measured by Medical Research Council scale), gastrointestinal features (gastroesophageal reflux disease, dysmotility of oesophagus, dysphagia and difficulty in swallowing), respiratory/cardiovascular manifestations (dry cough, shortness of breath on exertion), pericardial/myocardial involvement, and constitutional symptoms will be recorded. Pulmonary function tests, high resolution lung CT findings and cardiac ultrasound findings will be also obtained, upon clinical indication. Comorbidities including cancer, thyroid dysfunction, hypertension, coronary artery disease, and diabetes mellitus along with complete blood cell count, erythrocyte sedimentation rate, C-reactive protein, creatinine, liver function tests (transaminases, cholestatic enzymes), lactate dehydrogenase, creatinine phosphokinase, immunological tests (hypergammaglobulinemia, rheumatoid factor, anti-nuclear antibodies, complement levels), will also be documented. Serum and whole peripheral blood samples derived from patients and healthy controls will be collected and stored at −80°C. Written informed consent will be obtained from all participants.

### Autoantibody detection by immunoblot

Detection of MSAs/MAAs will be performed in serum samples collected from patients fulfilling the 2017 European League Against Rheumatism/American College of Rheumatology Classification Criteria for Adult and Juvenile Idiopathic Inflammatory Myopathies and Their Major Subgroups.^2^ More specifically reactivities against Mi-2a, Mi-2b, TIF-1γ MDA-5, NXP-2, SAE-1, Ku, PM/Scl-100-75, Jo-1, SRP, PL-7, PL-12, EJ, OJ, Ro-52, HMGCR, cN1A using the Euroline Autoimmune Inflammatory Myopathies (EUROIMMUN Medizinische Labordiagnostika AG) test kit following the manufacturer’s instructions will be sought.

### RNA extraction and gene expression analysis

Whole RNA extraction from patients’ and healthy controls’ peripheral blood will be performed using the TRIZOL reagent (Ambion/Thermo Fisher Scientific) according to manufacturer’s protocol. All samples will be treated with DNAse I (Qiagen, Germany) prior to cDNA synthesis. The quantity and quality of RNA samples will be spectrophotometrically tested (BiospecNano, Japan). Reverse transcription into cDNA of 0,5μg of whole RNA will be conducted using reverse transcriptase (SUPERSCRIPT III, Invitrogen/Thermo Fisher Scientific). Quantitative Real-Time Polymerase Chain Reaction(qRT-PCR) will be used to quantify specific cDNAs using the Bio-Rad IQ5 thermocycler and the IQ Bio-RadSYBR Green Supermix (Bio-Rad, Hercules, CA). Type I IFN inducible gene expression will assessed as previously described.^13^

### DNA extraction

DNA extraction from patients’ and healthy controls’ peripheral blood will be performed using commercially available reagent Quick-DNA Mini-Prep Plus kit (Zymo Research, USA) following manufacturer’s instructions.

### Flow cytometry

Sialic Acid Binding Ig Like Lectin 1 (SIGLEC1) protein expression will be assessed in the peripheral blood of patients and controls using flow cytometry technique at the CyFlow ML PARTEC cytometer as previously described.^21^

### Statistical analysis

Statistical analysis will be performed by SPSS v.26 package. Two-group comparisons of continuous data will be assessed using t-tests, or the Mann-Whitney test in case of non-Gaussian data distribution. Comparisons between groups will be performed by Fisher’s exact two tailed test and Mann Whitney test. Difference will be considered statistically significant, if p<0.05.

### Significance of the study

Taking into consideration the differential responsiveness to immunosuppressive therapies among IIM patients, the elucidation of the underlying immune pathways of diverse IIM subtypes could lead to more targeted and personalised therapeutic strategies.

## CONFLICT OF INTEREST

The authors declare no conflict of interest.
